# Usefulness of peripapillary nerve fiber layer thickness assessed by optical coherence tomography as a biomarker for Alzheimer’s disease

**DOI:** 10.1038/s41598-018-34577-3

**Published:** 2018-11-05

**Authors:** Domingo Sánchez, Miguel Castilla-Marti, Octavio Rodríguez-Gómez, Sergi Valero, Albert Piferrer, Gabriel Martínez, Joan Martínez, Judit Serra, Sonia Moreno-Grau, Begoña Hernández-Olasagarre, Itziar De Rojas, Isabel Hernández, Carla Abdelnour, Maitée Rosende-Roca, Liliana Vargas, Ana Mauleón, Miguel A. Santos-Santos, Montserrat Alegret, Gemma Ortega, Ana Espinosa, Alba Pérez-Cordón, Ángela Sanabria, Andrea Ciudin, Rafael Simó, Cristina Hernández, Pablo Villoslada, Agustín Ruiz, Lluís Tàrraga, Mercè Boada

**Affiliations:** 1 Alzheimer Research Center and Memory Clinic of Fundació ACE, Institut Català de Neurociències Aplicades, Barcelona, Spain; 2 Clínica Oftalmológica Dr. Castilla, Barcelona, Spain; 3 Valles Ophthalmology Research, Hospital General de Catalunya, Sant Cugat del Vallès, Spain; 4 Psychiatry Department, Hospital Universitari Vall d’Hebron, CIBERSAM, Universitat Autònoma de Barcelona, Barcelona, Spain; 5 Topcon España Clinical Affairs, Sant Just Desvern, Spain; 6 Faculty of Medicine and Dentistry. Faculty of Medicine and Dentistry, Universidad de Antofagasta, Antofagasta, Chile; 7 Iberoamerican Cochrane Centre, Barcelona, Spain; 8 Diabetes and Metabolism Research Unit and Centro de Investigación Biomédica en Red de Diabetes y Enfermedades Metabólica Asociada (CIBERDEM), Vall d’Hebron Research Institute, Barcelona, Spain; 9 Institut d’Investigacions Biomèdiques August Pi Sunyer (IDIBAPS), Barcelona, Spain

**Keywords:** Retina, Alzheimer's disease

## Abstract

The use of optical coherence tomography (OCT) has been suggested as a potential biomarker for Alzheimer’s Disease based on previously reported thinning of the retinal nerve fiber layer (RNFL) in Alzheimer’s disease’s (AD) and Mild Cognitive Impairment (MCI). However, other studies have not shown such results. 930 individuals (414 cognitively healthy individuals, 192 probable amnestic MCI and 324 probable AD) attending a memory clinic were consecutively included and underwent spectral domain OCT (Maestro, Topcon) examinations to assess differences in peripapillary RNFL thickness, using a design of high ecological validity. Adjustment by age, education, sex and OCT image quality was performed. We found a non-significant decrease in mean RNFL thickness as follows: control group: 100,20 ± 14,60 µm, MCI group: 98,54 ± 14,43 µm and AD group: 96,61 ± 15,27 µm. The multivariate adjusted analysis revealed no significant differences in mean overall (p = 0.352), temporal (p = 0,119), nasal (p = 0,151), superior (p = 0,435) or inferior (p = 0,825) quadrants between AD, MCI and control groups. These results do not support the usefulness of peripapillary RNFL analysis as a marker of cognitive impairment or in discriminating between cognitive groups. The analysis of other OCT measurements in other retinal areas and layers as biomarkers for AD should be tested further.

## Introduction

Alzheimer’s disease (AD) is a complex neurodegenerative disease and the most common cause of dementia^1^. Clinical diagnostic criteria for AD do not discriminate with accuracy between different dementing etiologies^2^. Before the onset of dementia, cognitive disorders progress slowly with minor cognitive impairment and without significant interference in daily activities. This prodromal phase is known as mild cognitive impairment (MCI), a clinically heterogeneous syndrome whose definition has evolved in last years^3–5^ and can be due to many different etiologies (AD, vascular damage, depression,…). Although some MCI patients can remain stable for decades or even return to cognitive normality, it is well established that amnestic and multi-domain MCI condition increases the risk of progressing to AD^6,7^. Given the fact that diagnosis of AD is still complicated especially in the MCI stage, the search of inexpensive and noninvasive biomarkers is a promising area of research^8^. Even though some biomarkers have been validated and integrated into the new clinical diagnostic criteria^9,10^, most show suboptimal test accuracy and either are very expensive such as detection of Aβ and Tau deposits in the brain using positron emission tomography (PET) or fairly invasive such as measurement of tau protein and Aβ peptide levels in cerebrospinal fluid (CSF) analysis^11,12^

The retina is considered an anatomical protrusion of the central nervous system with same embryological origins. Unmyelinated axons of retinal ganglion cells form the retinal nerve fiber layer (RNFL) which prolongs as the optic nerve and connects to the lateral geniculate nucleus (LGN) in the thalamus, which serves as the first relay center of the visual pathway.

Color and contrast sensitivity impairment, worse depth and motion perception, visual field deficits and impaired visual acuity are often seen in AD patients^13,14^. Visual symptoms in AD are supposedly caused by damage to associative visual cortical areas^15,16^, however, there is mounting evidence that neuroretinal involvement could also be a contributing factor^17^ and this has spurred interest in the search for retinal AD biomarkers^18,19^. Numerous postmortem histopathological studies reported RNFL and ganglion cell layer (GCL) reduction in AD patients^18,19^ although others demonstrated disparate results^20–22^.

Optical coherence tomography (OCT) is a relatively inexpensive, innocuous, quick transpupillary technique that permits *in vivo* objective retinal measurements and quantification. OCT is routinely used in ophthalmology to evaluate retinal integrity through high-resolution cross-sectional scans of retinal layers such as the RNFL and CGL at different locations such as the macula or papilla^23,24^. OCT is widely used in clinical ophthalmology and is a promising tool for neurological research^25^ due to its good reliability in a variety of Central Nervous System (CNS) pathologies^26–28^ and high correlation with several visual electrophysiological techniques^29,30^.

Peripapillary RNFL thinning is the most common finding in many neurological conditions such as multiple sclerosis, stroke, neuromyelitis optica, Lewy Body Dementia, Parkinson’s disease and AD^31–33^. It has been postulated to occur due to retrograde degeneration of the retinal ganglion cell axons or retinal deposits of AD pathology. Of note, classical histological studies^18,19^ have not found beta-amyloid plaques or neurofibrillary tangles in the retina of AD patients but more recent studies claim to detect them^34–36^.

Studies on peripapillary RNFL are inconclusive as they are based on small size samples and show important methodological heterogeneity^37–39^ and discrepant results as will be discussed in further sections. Some studies show marked RNFL thinning affecting all retinal quadrants in patients with AD and MCI^13,40–43^, whereas others do not^26,44–46^.

Our study aims to assess the clinical usefulness and feasibility of collecting RNFL thickness using OCT in a memory unit (MU) from a large, consecutively recruited cohort.

## Results

**Figure 1:**
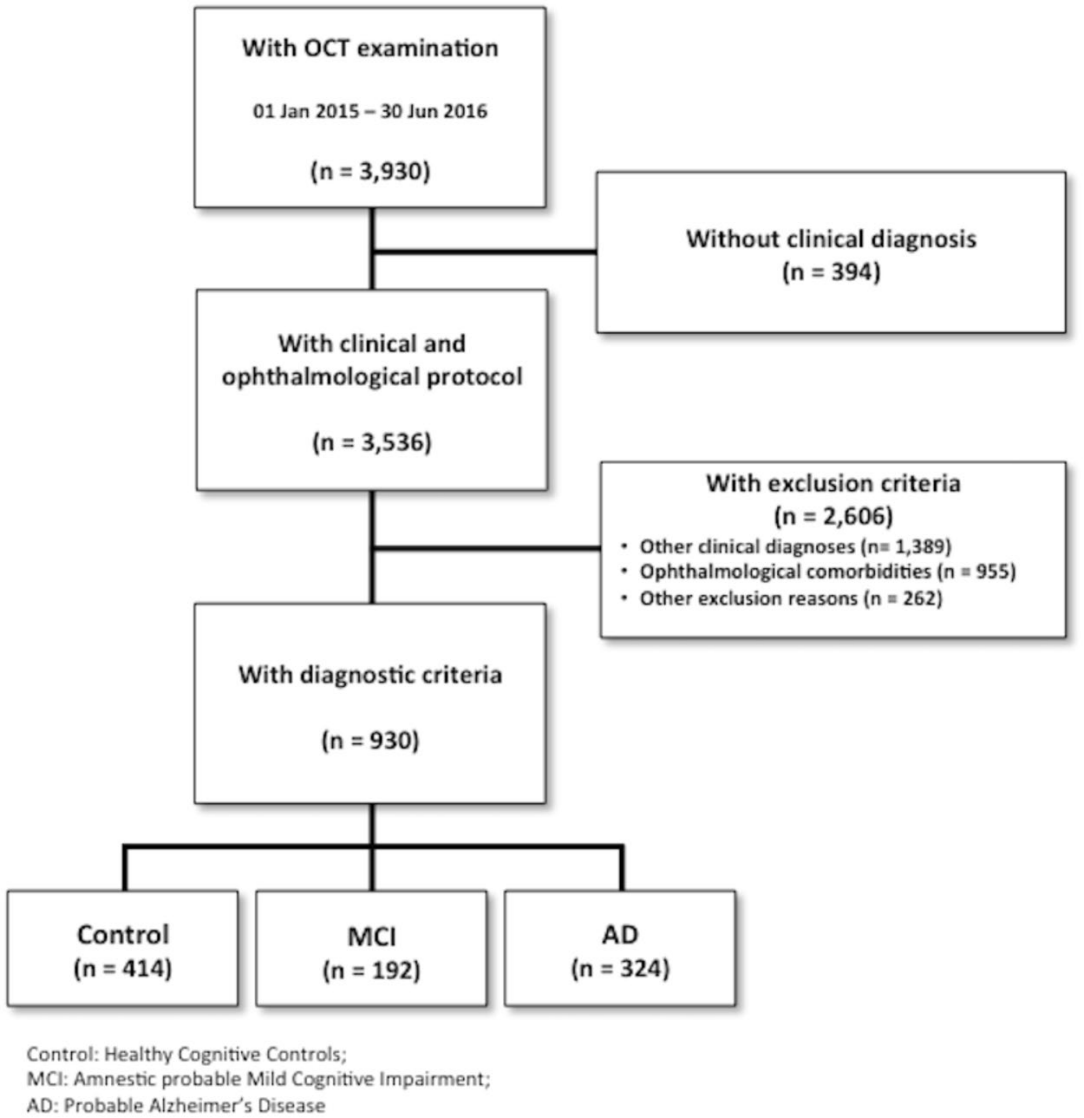
Patient selection and study cohort flow chart. Eligible population and selection of the study sample for this study through inclusion and exclusion criteria.

Figure 1 depicts the participant algorithm selection. 3,930 subjects attending a MU from January 2015 to July 2016 were invited to take part in this study and underwent a complete ophthalmological examination. 3,536 individuals (90%) received clinical diagnosis. 955 (24.4%) individuals out of 3,930 patients were discarded owing to several eye diseases (Table 1). Glaucoma and degenerative maculopathy were the main reasons for exclusion and their prevalence was especially high, especially among older and AD patients.

**Table 1:** Ophthalmological causes of exclusion.

Causes	Number (% over excluded)	Prevalence in MU
Glaucoma	292 (30.6)	7,4%
Macular degeneration	289 (30.3)	7,3%
Amblyopia	135 (14.1)	3.4%
Intraocular pressure >24	62 (6.5)	1.6%
Retinal surgery	46 (4.8)	1.1%
OCT artifacts	39 (4.1)	1.0%
Myopia magna	36 (3.8)	0.9%
Optic neuropathies	18 (1.9)	0.5%
Ocular injury	6 (0.6)	0.2%
Other causes	61 (6.4)	1.6%
Unknown	72 (7.6)	1.8%

955 out of 3,930 participants were excluded because of ophthalmological causes. Some subjects met more than one ophthalmological exclusion criterion.

After the comprehensive application of inclusion and exclusion criteria depicted in detail below (see Methods), 930 (23.6%) participants in the cohort met all the inclusion criteria and none of the exclusion criteria: 414 subjects were in the control group, 192 in the MCI group and 324 in the AD group. Demographics are described in Table 2.

**Table 2:** Baseline demographics.

		Mean	SD	Inter-groups Significance
Education (years)	Control	10.96	4.13	
	MCI	7.00	4.32	
	AD	6.14	4.08	
	Total	8.46	4.72	<0.001^+^
Age (years)	Control	65.93	9.01	
	MCI	76.46	7.14	
	AD	78.99	7.87	
	Total	73.05	10.23	<0.001^+^
MMSE (points)	Control	29.29	1.00	
	MCI	25.14	2.97	
	AD	20.28	3.98	
OCT Image Quality (%)	Control	47.81	7.57	
	MCI	44.59	8.23	
	AD	43.23	10.33	
	Total	45.41	9.09	<0.001^+^
Gender (% women)	Control	67.7%		
	MCI	56.2%		
	AD	74.0%		
	Total	67.8%		<0.001*

Demographic features like age, gender and education, MMSE scores, and OCT quality image among groups are summarized. All the analyzed characteristics were significantly different among diagnostic groups.

^*^Pearson’s Chi^2^ test.

^+^1-factor ANOVA.

There were significant differences between all three diagnostic groups in all demographical features (p < 0.001). Women made up most of the total sample and were significantly more prevalent in the AD than in the MCI group. Cognitively healthy individuals were younger and showed higher education levels, higher MMSE scores and a slightly better OCT image quality than both MCI and AD patients. The MCI group was younger and scored higher in the MMSE (Mini-mental State Examination) than the AD group.

Table 3 summarizes each covariate’s contribution to RNFL thickness variance including eta-squared and significance, after using ANCOVA.

**Table 3:** Contribution of every covariate to variance of RNFL thickness.

Covariate	Significance	*D.f.	Partial Eta^2^
Education	0.018	1	0.006
Gender	0.516	1	0.000
Age	0.0001	1	0.033
OCT image quality	0.0001	1	0.037
Diagnosis	0.352	2	0.002

Correlation between demographical and opthalmical covariates with the dependent variable is itemized.

*D.f.: Degrees of Freedom.

OCT image quality, followed by age and education turned out to be the most decisive factors for explaining variability of RFNL thickness and showed a very significant correlation and effect size. These three variables were more strongly associated with RNFL measurements than the diagnosis itself, whose correlation was not significant. The effect of gender was not significantly correlated with the peripapillary RNFL thickness.

Due to the differences in demographics between groups, we adjusted overall and sector-specific RNFL thickness in a multivariate model by the following covariates: OCT image quality, age, gender and education. No significant differences in mean overall (p = 0.352), temporal (p = 0.119), nasal (p = 0.151), superior (p = 0.435) and inferior (p = 0.825) RNFL thickness were found after comparing diagnostic groups. Our Mean and SD values are within the range of previously published studies.

**Figure 2:**
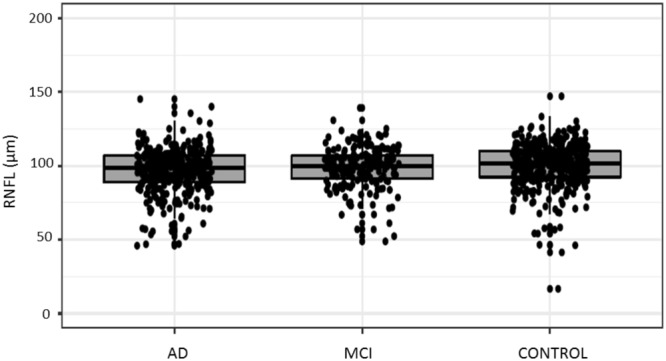
Mean RNFL for each diagnostic group. This boxplot represents the mean RNFL for each diagnostic group (Control, MCI, AD). No differences were found between clinical categories. The bottom and top of the box are the first and third quartiles and the band inside the box represents the median. AD: Alzheimer’s Disease; MCI: Mild Cognitive Impairment.

Raw and adjusted data is displayed for mean overall (Table 4) and sector-specific (Table 5) RNFL thickness. A boxplot for mean overall RNFL thickness is represented (Fig. 2).

**Table 4:** Results of mean RNFL in all diagnostic groups.

Group (N)	Mean	SD	Mean^aa^	SEM	p value
Control (414)	100.20	14.59	97.81^aa^	0.82	
MCI (192)	98.54	14.43	99.86^aa^	1.05	
AD (324)	96.61	15.27	98.88^aa^	0.89	
Total (930)	—	—		—	0.352

Raw and adjusted mean overall RNFL thickness (μm), standard deviation (SD) and standard error of the mean (SEM). After a multivariate adjustment, no significance between any diagnostic groups appeared and dispersion data is shown as SEM.

SD: Standard Deviation; ^aa^after adjustment; SEM: Standard Error of the Mean; p: Significance; AD: Alzheimer’s Disease; MCI: Mild Cognitive Impairment.

**Table 5:** Results of quadrants RNFL in all diagnostic groups.

p value	Group (N)	Mean	SD	Mean^aa^	SEM
Temporal 0.119	Control (413)	72.76	12.43	71.44^aa^	0.77
	MCI (192)	72.46	13.73	73.24^aa^	0.99
	AD (324)	72.82	14.63	74.04^aa^	0.83
	Total (929)		—	—	—
Superior 0.435	Control (413)	117.74	24.54	114.88^aa^	1.34
	MCI (192)	116.31	24.17	117.74^aa^	1.73
	AD (324)	114.27	22.34	117.05^aa^	1.45
	Total (929)		—	—	—
Nasal 0.151	Control (413)	78.67	18.18	77.12^aa^	1.00
	MCI (192)	78.95	17.27	79.68^aa^	1.29
	AD (324)	75.18	17.65	76.72^aa^	1.08
	Total (929)		—	—	—
Inferior0.82 5	Control (413)	131.80	21.97	128.06^aa^	1.28
	MCI (192)	126.34	21.82	128.53^aa^	1.65
	AD (324)	123.82	25.25	127.28^aa^	1.39
	Total (929)		—	—	—

Raw and adjusted sectorial RNFL thickness (μm), standard deviation (SD) and standard error of the mean (SEM). After a multivariate adjustment, no significance between any diagnostic groups appeared and dispersion is shown as SEM.

SD: Standard Deviation; ^aa^after adjustment; SEM: Standard Error of the Mean; p: Significance (missing data: 1); AD: Alzheimer’s Disease; MCI: Mild Cognitive Impairment.

Since previous studies showing significant differences in RNFL thickness compared data between age and gender matched groups instead of using multivariate regression, we performed a sensitivity analysis in some age and gender groups at different age intervals to verify that the observed lack of significance in RNFL reduction between diagnostic groups was not due to this methodological difference. No significant differences were obtained by comparing age and gender matched groups (Table 6).

**Table 6:** Example of mean RNFL thickness in all diagnostic groups using a matching strategy for age.

Group (N)	Women (%)	p value	Age (years)	SD	p value
Control(85)	55.3		74.48	2.87	
MCI (84)	58.3		74.55	2.64	
AD (63)	63.5		74.81	2.61	
Total (232)	58.6	0.60	74.62	2.70	0.99
**Group (N)**	**p RNFL**	**SD**	**p value**		
Control (85)	97.36	13.03			
MCI (84)	100.44	15.10			
AD (63)	99.88	12.33			
Total (232)	99.16	13.65	0.185		

Mean age (years), mean peripapillary RNFL thickness (μm) and standard deviation (SD) are shown. Age (p = 0.99) and gender (p = 0.60) do not differ among all diagnostic groups. Changes in RNFL thickness between any diagnostic groups were not significant (p = 0.185).

SD: Standard Deviation; p: Significance; p RNFL: Peripapillary RNFL; AD: Alzheimer’s Disease; MCI: Mild Cognitive Impairment.

## Discussion

In contrast to some previous studies, we have not found significant differences in peripapillary RNFL thickness between cognitively healthy subjects, MCI and AD patients. However, previous literature on this matter is discrepant and inconclusive so far. Most publications showed that peripapillary RNFL thinning might affect all quadrants in the AD as well as in the MCI stage and correlates with functional measurements of visual pathways^13,40–43^. However, other recent studies do not show a marked reduction of disc RNFL thickness^26,44–46^. Such discrepancies may be due to several reasons such as lack of a strong involvement of the RNFL in AD, and, therefore, the effect is not detectable.

Due to the difficulty to recruit very old cognitive controls and very young people with dementia, the examined age interval in previous literature was very reduced (mean age between 70 and 80 years). Thus, cognitive impairment happening at very early or very advanced ages was not taken into account and it is known that rates of cognitive decline vary depending on the age^47^. Other important factors affecting cognition such as education or affecting measurements such as OCT image quality were seldom studied in prior investigations. Our study tests both of these key covariates and in all age intervals showing that OCT image quality appears to be the most influential covariate determining peripapillary RNFL variability, followed by age. OCT signal strength is considered as a proxy of OCT image quality. Its value ranges from 0 (minimal) to 1 (maximal) and is given automatically by the software, after each acquisition. Controls got higher OCT signal than AD patients. This parameter also appeared to be much more influential in OCT protocols assessing disc than macula possibly because the disc protocol requires excentrical fixation and therefore it is more demanding for the cognitively impaired patients than for healthy controls whose attention is better. It is also possible that cognitively impaired patients have more subclinical corneal or lens opacities than healthy individuals, even after having excluded all the subjects with evident ocular etiologies, due to the known comorbidity between AD and degenerative maculopathies^48,49^. Thus, we consider that image quality values should always be included in future research when studying the optic disc using OCT.

We have conducted a cross-sectional study using a large sample representative of clinical practice in a wide range of ages, cognitive stages and medical etiologies in order to enhance ecological validity. Most patients from our cohort were able to collaborate during OCT performance and this fact supports the feasibility of OCT assessment in a memory clinic setting. However, 24.4% of eligible subjects were excluded due to ophthalmological comorbidities, mainly glaucoma and macular degeneration. Both are diseases associated to age and different authors consider that both diseases share some pathophysiological underlying mechanisms with AD^48,49^ and it could partly explain the high prevalence observed in our cohort. The fact that these retinal pathologies also affect RNFL thickness might make the application of OCT as biomarker in a memory clinic more complex.

We consider our study counts with various strengths compared to previous investigations that study the retina in AD. Unlike other studies, where diagnostic criteria for cognitive normality and impairment are not detailed or only based on simple screening tests, we applied a comprehensive and standardized diagnostic protocol including extensive neuropsychological testing and neuroimaging biomarkers, as shown in the Methods. Most studies in the past were case-control designs based on small, hyperselected and underpowered sample sizes^37–39^. Their methods and results were very heterogeneous, given the fact that some studies included only one eye per patient whereas others included both, that different OCT techniques and brands were used, etcetera. Heterogeneity between studies can be measured through the Higgins I^2^ test^50^. I^2^ was calculated in three meta-analysis^37–39^ showing values over 95%, indicating an extreme lack of homogeneity that makes comparison difficult. Inclusion of participants in previous studies was not consecutive and investigators were not blinded to the clinical diagnosis before OCT execution leading to important risk of bias and overestimating test accuracy^51,52^. We took special care to avoid these limitations, by including every consecutive patient attending the MU, independently of their clinical presentation, education and age. The cohort of this study is the largest sample size collected in a single site so far. To avoid bias, clinician and optometrist were blinded to all procedures carried out in the same patient by the other. In our results, peripapillary RNFL is not discriminating enough between cognitive groups and its normal variability due to ageing and other factors might be fairly more pronounced than the variability hypothetically due to active neurodegeneration. Our results do not support the usefulness of RNFL thickness as biomarker of cognitive impairment in a MU.

We also acknowledge several limitations of this study. First of all, the results are only cross-sectional, therefore no conclusions can be drawn about the dynamics of RNFL thinning. We envision a longitudinal analysis to elucidate the RNFL changes over time. Second, standard deviation of RNFL measurements showed an important inter-individual variability within each group, similar to the one observed in some population-based or normative studies^53,54^. However, this higher variability may be related to the fact that the age range in our population is less restricted than in the case-control studies previously mentioned.. Third, given the heterogeneity of clinical and visual symptoms in AD, it may be possible that there are certain subgroups of AD patients with significant lesions of the visual system and this fact could contribute to explain observed discrepancies in the literature^45^. Fourth, the covariates considered in our model might not be sufficient to control for inter-group variability, as we did not consider important eye parameters that might be confounding factors such as axial length and optic disc area, among others^53^.

Although controversial, the peripapillary RNFL thinning is the most published retinal discovery in AD. However, many other OCT findings such as reduction in macular volume, ganglionar cell layer thickness, choroid width and some vascular alteration have been described and might be promising biomarkers for dementia staging and AD progression^55–59^. In fact, it is possible that retinal AD biomarkers can only be achieved after having integrated various of the already cited biomarkers, both neuroretinal (such as RNFL, CGL, macular width) and retinovascular parameters (vessel morphology among others), in a composite biomarker. In any case, rapid advancements both in OCT technology such as automatic segmentation and in biomarkers in PET/CSF to improve diagnostic certainty^60,61^, can give us a better insight into relationships between brain and eye.

## Methods

### Participant selection and characterization: the NORFACE cohort

The Neuro-Ophthalmology Research At Fundació ACE (NORFACE) research cohort was founded to search for retinal biomarkers of AD and examine the intriguing relationships between retinal pathophysiology and different types of neurodegenerative disease that cause cognitive impairment including AD, frontotemporal dementia, Lewy body dementia, Parkinson’s Disease and vascular dementia among others. It is characterized by (a) prospective and consecutive recruitment in a memory clinic (b) extensive neurological, neuropsychological and socio-functional evaluations (c) a complete neuro-ophthalmological evaluation including a OCT scan, (d) consensus-based clinical diagnosis made by a multidisciplinary team (e) a longitudinal periodical clinical and neuro-ophthalmological reassessment. Fundació ACE-Institut Català de Neurociències Aplicades, Barcelona, Spain has developed a multidisciplinary approach to diagnose and care for patients with neurodegenerative diseases^62^ based on standardized neuropsychological and medical examinations^63,64^. Patients are referred to Fundació ACE-Memory Unit (FACE-MU) by primary care physicians or medical specialists of the Catalan Public Health System and undergo cognitive and psychological screening, including the Mini-Mental State Examination (MMSE)^65,66^, the 7-minute tests^67^ and the Hospital Anxiety and Depression Scale (HAD)^68^. Afterwards, a comprehensive neuropsychological battery called NBACE is administered. NBACE counts with normative data and includes tests sensitive to orientation, memory, language, executive, visuoconstructive, visuospatial and visuoperceptive functions^63,64^.

Blood tests comprising syphilis screening, liver and renal function, cholesterol profile, thyroid function and serum vitamin B12 and folate levels are analyzed to exclude possible causes of dementia. Brain atrophy, in particular medial temporal lobe (MTL) involvement is assessed by structural MRI and CT to improve diagnostic certainty^69^. For some selected individuals PET or CSF data is also available^60,61^. After the neurologist’s and neuropsychologist’s clinical examination made by the neurologist, a consensus-based diagnosis is reached by a multidisciplinary team (neurologist, neuropsychologist, social worker).

Since January 2015 all new and follow-up patients attending the FACE-MU who are able to collaborate and understand instructions, undergo a complete a neuro-ophthalmological history and examination always by the same optometrist, before reaching the definite clinical diagnosis. The assessment includes (i) ophthalmological history, (ii) best-corrected visual acuity assessment by subjective refractometry using the Early Treatment of Diabetic Retinopathy Study (ETDRS) chart, (iii) slit lamp examination was only used if the optometrist considered that biomicroscopy/ophthalmoscopy would be useful based on the medical history or on any unexpected finding seen in OCT scans, (iv) intraocular pressure measurement by Icare tonometry (v) and spectral domain OCT examination.

The optometrist and neurologist were blinded to all data from each other evaluation.

### Optical Coherence Tomography

Patients were imaged with a 3D-OCT Maestro®, Fast Map software version 8.40 (Topcon Co. Tokio, Japan) to assess macular and optic disc regions. Importantly, no pupil dilatation was required because of high resolution Bscan mode which also allows to get also CScan confocal imaging through the EnFace software. The OCT capture is combined with a real color fundus picture obtained through an internal camera.

Retina layer segmentation was performed using the TABS (Topcon Advanced Boundary SegmentationTM) algorithm as part of the Fast Map software. TABS provide accurate and consistent measurements on retina images, improving reproducibility and accuracy together with great stability in performance across blood vessel shadows and it has proved effectiveness in ophthalmological diseases^70,71^. It also provides multi-layer delineation in one aggregate operation including the internal limiting membrane (ILM), nerve fiber layer (NFL), ganglion cells layer (GCL) boundary, inner plexiform layer (IPL), inner nuclear layer (INL) boundary, retinal pigment epithelium (RPE) and Bruch’s membrane (BM). NFL (from ILM to NFL/GCL), GCL+ (from NFL/GCL to IPL/GCL) and total retinal thickness (from ILM to RPE) was automatically generated by TABS. RNFL parameters evaluated in this study were average or mean overall thickness (360° measurement), temporal quadrant thickness (316–45°), superior quadrant thickness (46–135°), nasal quadrant thickness (136–225°) and inferior quadrant thickness (226–315°). For this study, OCT data was only analyzed from one eye (right). After finishing a OCT imaging session, the same optometrist screened all images searching for abnormalities and sent all images to a consultant ophthalmologist, an expert in retinal pathology, who reviewed them all, one by one and made a diagnostic report.

### Eligibility criteria

Patients were included if they were between 50 and 95 years of age and met control, MCI or AD diagnostic criteria described in continuation. The control group was defined by (a) absence of significant symptoms (CDR = 0) and (b) a normal age, gender, and education-adjusted performance on NBACE^63,64^. The MCI group was defined by (a) meeting Petersen criteria for amnestic MCI^72^ and (b) absence of significant signs of cerebrovascular or psychiatric disease. The last criterion was applied as described in previous publications^6,73^ to increase the probability of AD etiology. All subjects in the AD group met National Institute of Neurologic and Communicative Disorders and Stroke-Alzheimer’s Disease and Related Disorders Association (NINCDS- ADRDA) criteria for probable AD^74^. Note that the chosen inclusion criteria require the absence of other diseases capable of producing similar symptoms, resulting in a study cohort with high probability of “pure” AD etiology. In other words, patients with MCI or dementia and signs of non-AD etiologies (even if AD was also thought to be present, such as in cases of mixed AD and non AD etiology) were excluded to avoid confounding effects on the analysis of the relationship between RNFL thickness and AD-spectrum groups.

Patients were excluded if they could not understand or collaborate in the neuro-ophthalmological evaluation, if there was only data obtained from the left eye or presence of OCT artifacts or diseases that could affect OCT measurement such as glaucoma, maculopathies, neuropathies, prior retinal surgery, an intraocular pressure ≥ 24 mmHg, high myopia (<−6Dp), or hyperopia (>+6Dp), congenital abnormalities of the optic nerve. These excluded patients usually have low OCT signal strength but this parameter was not an exclusion criteria by itself.

### Ethical considerations

This study and its informed consent were approved by the ethics committees of both the Hospital Clínic I Provincial and the Hospital Vall D’Hebron, (Barcelona, Spain) in accordance with Spanish biomedical laws (Law 14/2007, July 3^rd^, about biomedical research; Royal Decree 1716/2011, November 18^th^) and followed the recommendations of the Declaration of Helsinki. All the participants signed the informed consent.

### Statistical analysis

**Figure 3:**
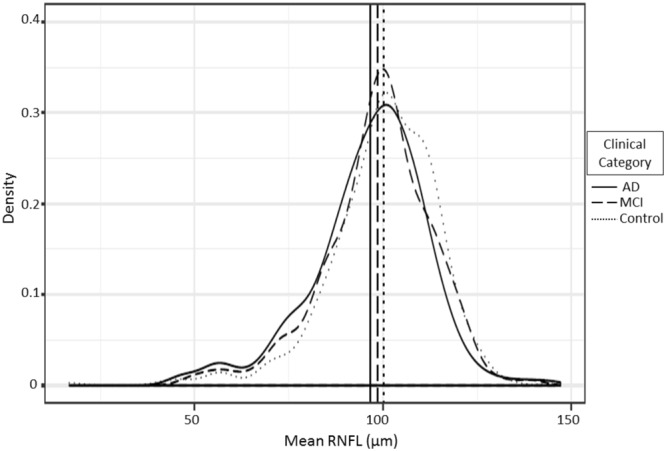
Mean RNFL distribution for all the diagnostic groups. AD: Alzheimer’s Disease; MCI: Mild Cognitive Impairment.

Statistical analyses were performed using IBM SPSS 20 (SPSS Inc., Chicago, IL) and according to APOSTEL guidelines^75^. All data were examined for normality, skew, and restriction of range. All quantitative variables were normally distributed (Fig. 3). The results are presented as mean ± SD for quantitative variables, range and number and percentage for categorical variables.

Chi-Squared test and parametric Student t-test were used to compare the demographic characteristics, clinical diagnoses and OCT measurements. The primary analysis of the study, assessing differences in the RNFL between subgroups adjusted by age, education, gender and image quality was tested with ANCOVA, with mean overall RNFL or sector-specific RNFL thickness (the sectorial areas were included in the same multivariate analysis, according to its correlated profile), as dependent factors and clinical groups as independent factors (three categories). Age, gender, years of education and OCT image quality were included in the model as adjustment variables. Eta2 was calculated for every factor of the model, obtaining the explained variance of all the predictors. A significant effect was considered when p < 0.05.

## Data Availability

The datasets generated during and/or analysed during the current study are available from the corresponding author on reasonable request.
