# Author Correction: Usefulness of peripapillary nerve fiber layer thickness assessed by optical coherence tomography as a biomarker for Alzheimer’s disease

**DOI:** 10.1038/s41598-019-53339-3

**Published:** 2019-11-08

**Authors:** Domingo Sánchez, Miguel Castilla-Marti, Octavio Rodríguez-Gómez, Sergi Valero, Albert Piferrer, Gabriel Martínez, Joan Martínez, Judit Serra, Sonia Moreno-Grau, Begoña Hernández-Olasagarre, Itziar De Rojas, Isabel Hernández, Carla Abdelnour, Maitée Rosende-Roca, Liliana Vargas, Ana Mauleón, Miguel A. Santos-Santos, Montserrat Alegret, Gemma Ortega, Ana Espinosa, Alba Pérez-Cordón, Ángela Sanabria, Andrea Ciudin, Rafael Simó, Cristina Hernández, Pablo Villoslada, Agustín Ruiz, Lluís Tàrraga, Mercè Boada

**Affiliations:** 1 Alzheimer Research Center and Memory Clinic of Fundació ACE, Institut Català de Neurociències Aplicades, Barcelona, Spain; 2 Clínica Oftalmológica Dr. Castilla, Barcelona, Spain; 3 Valles Ophthalmology Research, Hospital General de Catalunya, Sant Cugat del Vallès, Spain; 4 Psychiatry Department, Hospital Universitari Vall d’Hebron, CIBERSAM, Universitat Autònoma de Barcelona, Barcelona, Spain; 5 Topcon España Clinical Affairs, Sant Just Desvern, Spain; 6 Faculty of Medicine and Dentistry. Faculty of Medicine and Dentistry, Universidad de Antofagasta, Antofagasta, Chile; 7 Iberoamerican Cochrane Centre, Barcelona, Spain; 8 Diabetes and Metabolism Research Unit and Centro de Investigación Biomédica en Red de Diabetes y Enfermedades Metabólica Asociada (CIBERDEM), Vall d’Hebron Research Institute, Barcelona, Spain; 9 Institut d’Investigacions Biomèdiques August Pi Sunyer (IDIBAPS), Barcelona, Spain

Correction to: *Scientific Reports* 10.1038/s41598-018-34577-3, published online 05 November 2018

The original HTML and PDF version of this Article contained a typographical error in the spelling of the author Pablo Villoslada, which was incorrectly given as Pablo Villaoslada. This has now been corrected in the HTML and PDF versions of the Article.

